# The Medial Malleolar Fleck Sign: A Case Report Highlighting Retinacular Origin Rather Than Deltoid Ligament Avulsion

**DOI:** 10.7759/cureus.88210

**Published:** 2025-07-18

**Authors:** Yuko Yagi, Takeomi Nakamura

**Affiliations:** 1 Department of Spine and Orthopedic Surgery, Japanese Red Cross Medical Center, Tokyo, JPN; 2 Department of Orthopedics, Tokyo Metropolitan Hiroo Hospital, Tokyo, JPN; 3 Department of Orthopedics, Otakanomori Hospital, Chiba, JPN

**Keywords:** ankle fracture, ankle instability, avulsion fracture, computed tomography, deltoid ligament, extensor retinaculum, fixation, flexor retinaculum, medial clear space, medial malleolar fleck sign

## Abstract

The medial malleolar fleck sign (MMFS) is a radiographic indicator of medial ankle instability, conventionally interpreted as an avulsion of the deltoid ligament from its tibial insertion. However, its precise anatomical origin remains unclear, with limited intraoperative confirmation reported in the literature. We present a case of a 51-year-old woman with a left ankle inversion injury, resulting in a Weber B fibular fracture, posterior malleolar fracture, and a cortical fragment consistent with MMFS, accompanied by medial clear space widening. Intraoperatively, the deltoid ligament was ruptured but remained attached to its tibial origin. Notably, the MMFS fragment was avulsed from the tibial insertion of the flexor retinaculum (FR) and displaced anteriorly, with fibrous tissue extending forward, a finding highly suggestive of attachment from the extensor retinaculum (ER). The fragment was anatomically reduced and fixed, and the deltoid ligament was repaired. The patient regained full, pain-free function at one year. Given previous anatomical studies describing fascial continuity between the FR and ER, this case suggests a possible alternative mechanism of composite retinacular avulsion. These findings underscore that MMFS does not invariably indicate deltoid ligament injury, highlighting the importance of careful intraoperative assessment for accurate diagnosis and surgical planning.

## Introduction

The medial malleolar fleck sign (MMFS) is a small cortical avulsion fragment adjacent to the medial malleolus, often interpreted as a radiographic indicator of medial ankle instability [1]. It is commonly associated with supination-external rotation type ankle injuries, which are among the most frequent ankle fracture patterns [2]. It is typically presumed to reflect an avulsion of the deltoid ligament, a critical stabilizer on the medial side of the ankle. One clinical investigation reported that MMFS appeared in approximately 10% of ligamentous supination-external rotation fractures, demonstrating a high specificity of 99% and a positive predictive value of 94% for detecting instability. Notably, the sign remained highly specific even in cases without widening of the medial clear space (MCS), suggesting that it may indicate occult medial structural injury [1].

However, the precise anatomical origin of the fleck remains uncertain, with limited intraoperative confirmation reported in the literature. While often attributed solely to the deltoid ligament, other structures, such as the flexor and extensor retinaculum - bands of fibrous tissue that stabilize tendons around the ankle - also insert in this region and could potentially be involved in such avulsion injuries. This report presents a unique case in which intraoperative findings suggested that the fleck fragment originated not from the tibial attachment of the deltoid ligament but rather from the retinacular complex, specifically involving the flexor and extensor retinaculum, offering new insights into the potential anatomical sources of MMFS.

## Case presentation

**Figure 1:**
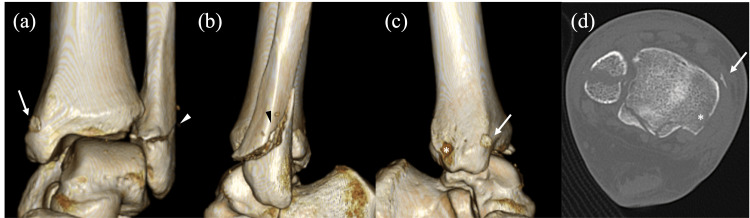
Preoperative computed tomography (CT) images of the ankle. (a-c) Three-dimensional reconstructed views. (a) Anterior view; (b) Lateral view; (c) Medial view; (d) Axial CT image. A cortical fragment consistent with the medial malleolar fleck sign (arrow) is seen anteriorly displaced from its avulsion site just proximal to the posterior colliculus (asterisk), along with a Weber B distal fibular fracture (arrowhead).

**Figure 2:**
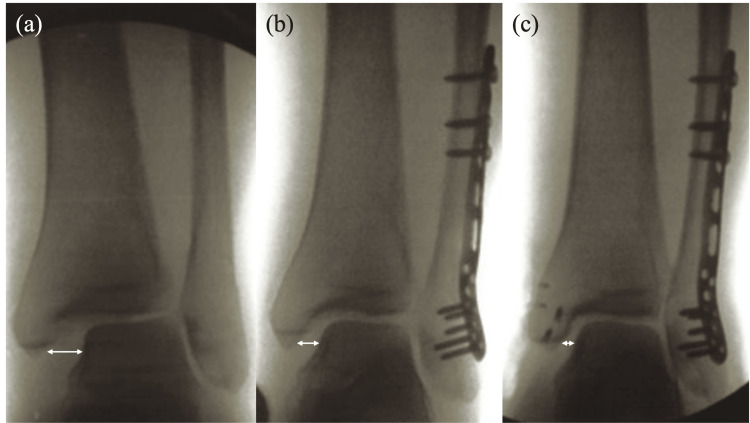
Intraoperative anteroposterior fluoroscopic views of the ankle. (a) Initial image showing widening of the medial clear space (MCS) (double arrow). (b) After fibular fixation, showing partial improvement of the MCS widening (double arrow). (c) After medial repair, showing restoration of the MCS (double arrow).

A 51-year-old woman sustained an inversion injury of her left ankle during a fall while walking. She was unable to bear weight and was transported to the hospital by ambulance. She had no prior medical conditions or a history of medication use. Physical examination revealed swelling and tenderness over both the medial and lateral aspects of the ankle. Radiographs demonstrated a Weber B distal fibular fracture, a posterior malleolar fracture, and a 6×6 mm cortical fragment just proximal to the posterior colliculus of the medial malleolus (Figures 1-2).

**Figure 3:**
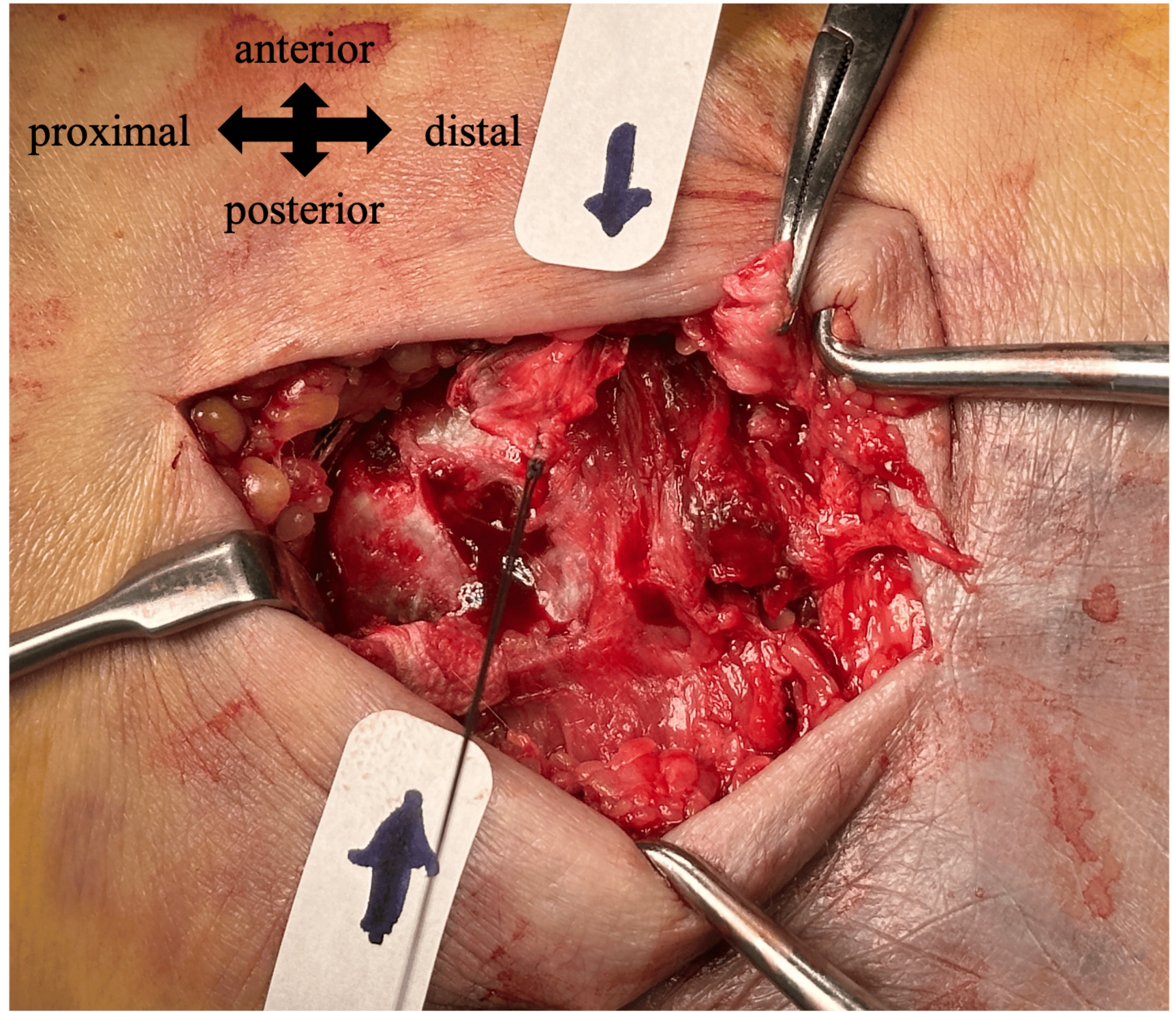
Intraoperative photograph of the medial malleolus. The avulsed cortical fragment (marked with sutures) is seen displaced anteriorly and separate from the torn deltoid ligament (grasped by forceps).

Widening of the MCS and distal tibiofibular diastasis were observed, consistent with ankle instability. On post-injury day 5, an open reduction and internal fixation procedure was performed. Through a lateral longitudinal incision, the fibular fracture was anatomically reduced and stabilized with a posterolateral locking compression plate (DePuy Synthes, West Chester, PA, USA). Although fibular fixation partially reduced the widening of the MCS and the distal tibiofibular joint, residual widening persisted on intraoperative fluoroscopy, and the hook test remained positive, prompting further medial exploration (Figure 2). Through a longitudinal incision centered over the medial malleolus, complete rupture of both superficial and deep components of the deltoid ligament was identified. A small cortical fragment was avulsed from the superior aspect of the posterior colliculus and was displaced anteriorly (Figure 3).

Notably, the deltoid ligament was not attached to the fragment. Instead, fibrous tissue extended anteriorly from the bony fragment, suggesting attachment from the extensor retinaculum (ER); no posteriorly extending fibers were identified.

The deltoid ligament was first reattached to its tibial insertion site using two suture anchors (Arthrex, Naples, FL, USA). Additionally, the remnant fibers were sutured together. The cortical fragment was reduced to its anatomical footprint and fixed with two suture anchors. The presumed retinacular fibers were reinforced to the periosteum with additional sutures. This combined repair resulted in the restoration of medial stability and correction of the widened MCS and distal tibiofibular joint, and resolution of instability as evidenced by a negative hook test (Figure 2).

Postoperative management included two weeks of immobilization, followed by the gradual initiation of range-of-motion exercises. Partial weight-bearing was introduced at week 3 and progressed to full weight-bearing by week 10. At the one-year follow-up, the patient remained pain-free and was able to ambulate without restriction. Ankle range of motion demonstrated 0° of dorsiflexion on the affected side and 5° on the contralateral side, with 35° of plantarflexion bilaterally. The patient’s American Orthopaedic Foot and Ankle Society Score was 100, indicating a favorable clinical outcome [3].

## Discussion

The MMFS is considered a particular radiographic indicator of medial ankle instability, likely reflecting complete deltoid ligament injury in the setting of supination-external rotation type ankle injuries [1]. However, the true origin of the fragment is rarely confirmed surgically, and few reports provide intraoperative correlation. In this case, the deltoid ligament was torn but not avulsed from its tibial attachment and did not connect to the cortical fragment. Instead, the fragment bore anteriorly directed fibrous tissue and was found to originate from the superior aspect of the posterior colliculus. Anatomically, this region corresponds to the tibial insertion of the flexor retinaculum (FR), which spans from the medial malleolus to the calcaneus and forms the roof of the tarsal tunnel [4].

Avulsion of the FR is a rare injury and has been described almost exclusively in association with posterior tibial tendon dislocation, where the cortical fragment is accompanied by fibrous tissue extending posteriorly, consistent with avulsion from the tibial attachment of the FR [5,6]. When FR avulsion occurs in conjunction with posterior tibial tendon dislocation, conservative treatment is often unsuccessful, and surgical intervention is typically required. Reported procedures include retinacular reattachment using suture anchors, primary repair, or reconstruction with periosteal or tendon sheath flaps, frequently combined with retromalleolar groove deepening to enhance tendon stability [5,6]. Surgical outcomes have generally been favorable, with excellent functional recovery and a low risk of recurrence. In the present case, however, the cortical fragment exhibited fibrous tissue extending anteriorly, and no posterior tibial tendon dislocation was observed, making isolated FR avulsion unlikely. Attention was thus directed to the ER, composed of the superior extensor retinaculum (SER) and inferior extensor retinaculum (IER). The SER is a transverse band extending between the distal tibia and fibula, while the IER is more variable and often presents Y- or cruciate-shaped configurations inserting onto the medial malleolus or tibia [7,8]. Although rupture of the ER in adults is extremely rare, one case involving lateral trauma and tibialis anterior tendon bowstringing has been reported [9]. In pediatric populations, avulsion of the SER at the fibular insertion has been associated with subperiosteal hematoma [10]. However, medial-sided avulsion at the ER insertion has not been previously described in the adult literature.

Anatomical and imaging studies have demonstrated that the FR and IER are frequently interconnected. Magnetic resonance imaging studies reveal the merging of the FR and IER in over 60% of cases, and cadaveric studies have confirmed the presence of fascial continuity and shared insertions [4,7,8]. These findings support the hypothesis that the cortical fragment, in this case, may reflect a composite avulsion from the FR insertion site influenced by tensile force transmitted via the IER. Such a mechanism could explain the anterior displacement of the fragment, even in the absence of tendon or direct deltoid involvement.

Surgical findings clarified that the deltoid ligament was torn but intact at its tibial origin. Fibular fixation alone did not restore stability, as MCS widening and distal tibiofibular diastasis persisted, and the hook test remained positive intraoperatively. Considering these findings, we elected to perform additional medial stabilization. Given that recent evidence suggests anatomic deltoid ligament repair is associated with significantly lower rates of syndesmotic malreduction and hardware removal compared to trans-syndesmotic fixation, we opted for direct deltoid ligament repair [11]. The deltoid ligament was reattached to its anatomical origin using suture anchors and reinforced by suturing the remnant fibers together. The cortical fragment, suspected to represent retinacular attachment, was anatomically reduced and secured with suture anchors, and the attached fibrous tissue was further reinforced to the periosteum. Following these procedures, MCS narrowing was confirmed, and the hook test became negative, indicating restored medial stability. Although fixation of the cortical fragment appeared to contribute to mechanical stability, whether it directly influenced functional recovery remains uncertain and warrants further investigation.

This case highlights the importance of careful intraoperative assessment when interpreting MMFS. Surgeons should be aware that the fleck fragment may not always reflect deltoid avulsion but could arise from a retinacular or fascial interface, particularly in the presence of anatomical variation and continuity among supporting structures. However, this observation is based on a single case and may reflect a rare anatomical variant, which limits the generalizability of our findings. Future anatomical and clinical studies involving larger case series are needed to validate this proposed mechanism and to clarify the prevalence and clinical relevance of retinacular involvement in MMFS.

## Conclusions

This case illustrates an atypical presentation of the MMFS, not resulting from an avulsion fracture of the deltoid ligament but rather likely originating at the tibial attachment of the FR. The cortical fragment was found anteriorly displaced, with fibrous tissue directed forward, suggesting traction from the ER. Intraoperative findings confirmed that the deltoid ligament was torn but not avulsed and that the small bone fragment likely reflected retinacular involvement rather than ligamentous avulsion.

Anatomical studies have shown considerable variability and interconnection among ankle fascial structures, particularly between the FR and IER. This case supports the possibility that the bone fragment resulted from an avulsion of the retinaculum at its tibial attachment on the medial malleolus, mediated through fascial continuity, which is distinct from classic deltoid avulsion patterns. However, this conclusion is based on a single case and may reflect a rare anatomical variant. Further anatomical and clinical research is needed to clarify the spectrum of MMFS origins. Nevertheless, careful consideration of the anatomical origin of the bone fragment is essential for accurate diagnosis and selection of an appropriate treatment strategy in cases presenting with the MMFS.

## References

[1] Nwosu K, Schneiderman BA, Shymon SJ, Harris T (2018). A medial malleolar "fleck sign" may predict ankle instability in ligamentous supination external rotation ankle fractures. Foot Ankle Spec.

[2] Jensen SL, Andresen BK, Mencke S, Nielsen PT (1998). Epidemiology of ankle fractures. A prospective population-based study of 212 cases in Aalborg, Denmark. Acta Orthop Scand.

[3] Kitaoka HB, Alexander IJ, Adelaar RS, Nunley JA, Myerson MS, Sanders M (1994). Clinical rating systems for the ankle-hindfoot, midfoot, hallux, and lesser toes. Foot Ankle Int.

[4] Szaro P, Gataa KG, Polaczek M, Ciszek B (2020). The flexor retinaculum connects the surrounding structures into the medial ankle complex. Appl Sci.

[5] Strydom A, Saragas NP, Tladi M, Ferrao PN (2017). Tibialis posterior tendon dislocation: a review and suggested classification. J Foot Ankle Surg.

[6] Al Khudairy A, Zafar MM, Padinjarathala BA (2013). The unexpected with ankle fracture: traumatic tibialis posterior tendon dislocation: a case report and literature review. Foot Ankle Spec.

[7] Abu-Hijleh MF, Harris PF (2007). Deep fascia on the dorsum of the ankle and foot: extensor retinacula revisited. Clin Anat.

[8] Shenoy MP, Pai MM, Murlimanju BV, Vadgaonkar R, Prabhu LV, Prameela MD (2025). Revisiting the anatomy of inferior extensor retinaculum of foot and ankle: a study based on fifty embalmed adult cadaveric lower extremities. Transl Res Anat.

[9] Tytherleigh-Strong G, Baxandall R, Unwin A (2000). Rupture of the ankle extensor retinaculum in a dancer. J R Soc Med.

[10] Ding J, Moraux A, Nectoux É, Demondion X, Amzallag-Bellenger É, Boutry N (2016). Traumatic avulsion of the superior extensor retinaculum of the ankle as a cause of subperiosteal haematoma of the distal fibula in children. A retrospective study of 7 cases. Skeletal Radiol.

[11] Sogard O, McDonald J, Waters ME, Lee W (2025). The clinical outcome comparison between trans-syndesmotic fixation and anatomic deltoid ligament repair in unstable ankle fractures with medial clear space widening: a systematic review and meta-analysis. Foot Ankle Surg.

